# Detection of Sleeve Grouting Compactness Based on Acoustic Emission Technology

**DOI:** 10.3390/ma16041455

**Published:** 2023-02-09

**Authors:** Aiping Yu, Xianghao Li, Feng Fu, Xuandong Chen, Yan Zhang

**Affiliations:** 1 College of Civil and Architecture Engineering, Guilin University of Technology, Guilin 541004, China; 2 Guangxi Key Laboratory of New Energy and Building Energy Savin, Guilin University of Technology, Guilin 541004, China; 3 Collaborative Innovation Center for Exploration of Nonferrous Metal Deposits and Efficient Utilization of Resources, Guilin 541004, China; 4 Guangxi Engineering and Technology Center for Utilization of Industrial Waste Residue in Building Materials, Guilin 541004, China; 5 Department of Civil Engineering, School of Mathematics, Computer Science & Engineering, City, University of London, Northampton Square, London EC1V 0HB, UK

**Keywords:** acoustic emission, signal generator, elastic wave, grout sleeve, grouting compactness

## Abstract

Sleeve grouting compactness has a significant effect on the mechanical properties of rebar connections. However, a detection method for the grouting compactness inside the sleeve is still lacking. Therefore, the aim of this paper is to propose a new acoustic emission (AE) detection technology for horizontal defects and vertical defects in sleeves with different grout compactness. The basic waveform characteristic of the AE signal is analyzed. The results show that the count of acoustic emission signals decreases with the increase of grouting compactness, and the reduction rate of vertical defects is larger than that of horizontal defects. The acoustic emission waveform is further processed through wavelet packet decomposition. It is found that with the increase of grouting compactness, the composition of approximately 125–187.5 kHz in the signal is accelerated to approximately 62.5–125 kHz. The grouting compactness index is constructed by wavelet packet energy ratio. With the increase of grouting compactness, the compactness index decreases exponentially, indicating that the presence of defects can greatly reduce the attenuation of elastic wave energy. The compactness index is highly consistent with the size of defects and has little relationship with the distribution of grout materials. Experiments show that the proposed method is effective when grout defects reach a certain degree and provides a new method for sleeve grouting compactness detection.

## 1. Introduction

Modular buildings have attracted much attention due to their advantages such as fast construction speed, small restriction by natural conditions, controllable construction cycle, effective labor and material saving, energy consumption reduction and pollution reduction [1,2]. The effective connection between prefabricated components has been the focus of many researchers and it is also the basic condition for the popularization and application of reinforced concrete prefabricated buildings. Grout sleeve connection, because of its good seismic and bearing performance [3,4], is currently the main connection mode of prefabricated components used in reinforced concrete prefabricated buildings [5]. However, under the complex construction conditions on site, grout quality problems such as less grout and leakage often occur in the grout sleeve, which leads to the insufficient grouting of the sleeve. This will undoubtedly reduce the anchorage length of the internal steel bar and the grout material of the sleeve, thus affecting the stability and seismic performance of the structure [6,7]. Studies [8,9] show that the tensile strength, steel bar sliding amount, deformation performance and damage form of the sleeve are greatly affected by the grout saturation, which shows that the influence of the grouting compactness on the overall performance of the sleeve members cannot be ignored. Therefore, the identification and determination of the degree of infill in the grout sleeve is one of the key issues that needs to be solved urgently for the connection performance evaluation of the grout sleeve and even the popularization and application of reinforced concrete assembly.

In recent years, many scholars have carried out attempts and explorations for the detection and identification of sleeve grouting compactness. Due to the hidden characteristics of grout saturation, the detection and identification are mostly based on non-destructive testing technology. Ultrasonic [10,11] is a common detection method. The saturation of the grout sleeve is judged by the change of amplitude, velocity or frequency of the received signal. However, due to the serious attenuation and distortion of the waveform during the propagation of the ultrasonic wave, it is easy to cause errors in the detection results by using single parameters such as waveform amplitude and velocity to judge the saturation of the grout sleeve. The impact echo method [12,13] judges the internal defects of the object according to the difference between the reflection frequency of the defect surface and the air interface, but it is difficult to distinguish the location of the defects for the double-layer grout sleeve. The cross-sectional images of the structure can be obtained by industrial CT (Computed Tomography) [14] and X-ray [15], which can intuitively define the grout compact area and void area, but X-rays are harmful to the human body and equipment is expensive. The pre-embedded wire drawing method [16] can reflect whether the sleeve grout is full through the drawing strength of the pre-embedded wire, but it can only determine the density of the grout material at the outlet and cannot determine the internal density of the sleeve. Moreover, the outlet needs to be filled twice after the wire drawing. The embedded damping vibration sensor method [17] can detect the grout quality in the construction process, but the grout material remaining on the sensor can cause misjudgment. The piezoelectric impedance method [18] and the piezoelectric wave method [19] are tested by manually pasting piezoelectric patches on the specimen, and the piezoelectric ceramic material of the piezoelectric patch is small and fragile, so its accuracy is affected by artificial bonding technology. In addition, there are differences in the performance of different piezoelectric patches and there may be a certain degree of misjudgment. Based on previous studies, it can be found that the identification and evaluation of grouting compactness needs to be further studied. The method of detecting the effectiveness of identification and the simplicity of operation still needs further exploration and research.

Acoustic emission technology is an effective dynamic monitoring method. In recent years, it has gradually become one of the conventional nondestructive testing methods and is widely used in many research fields [20,21,22,23,24]. In civil engineering, acoustic emission technology is mostly used to monitor and analyze the size, location and development trend of internal damage of concrete materials or structures [25,26,27]. Some scholars try to use acoustic emission technology to study the mechanical properties of grout sleeve connection, which has achieved the expected results [28,29]. Based on this, scholars tried to use acoustic emission technology to detect the grouting compactness and they found the change pattern of acoustic emission signal parameters in three conditions of grout, grout void and grouting compactness [30,31,32], which shows that it is feasible to apply acoustic emission technology to the detection of grouting sleeve. However, the further quantitative analysis and the identification method of the grouting fullness have not been found in relevant studies. So, in this paper, using a signal generator as the excitation source combined with acoustic emission technology to detect the grouting compactness, the acoustic emission signal characteristic parameters and the waveform of different grouting compactness are analyzed, and the evaluation index of grouting compactness is defined by using the energy proportion of wavelet packet decomposition. The relationship model between grouting compactness and acoustic emission parameters is established. 

## 2. Experimental Test

### 2.1. Setting and Fabrication of Sleeve Grout Defects

**Figure 1:**
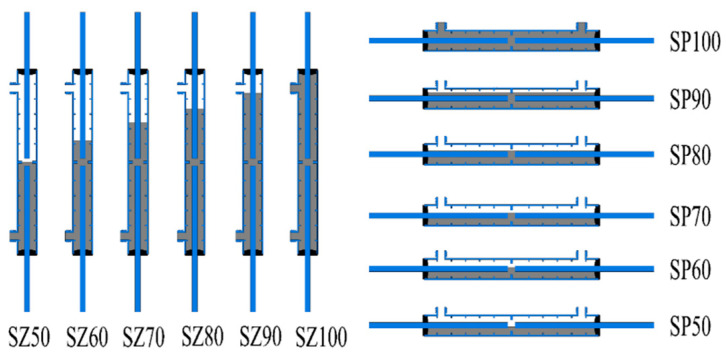
Schematic diagram of each saturation sleeve.

The purpose of this experiment is to verify the feasibility of the detection method based on acoustic emission technology. The process is to artificially produce grout defects first and then detect and verify the grouting compactness with an acoustic emission instrument. Therefore, it is necessary to artificially build a sleeve before grout with the actual size of a real project. Considering that the connection of longitudinal reinforcement in the beam is horizontally placed, there will be void defects of horizontal distribution in the sleeve caused by unfilled or leakage of grout material from the grout mouth, and the defects are set horizontally along the sleeve. Similarly, for the connection of the longitudinal reinforcement in the column, the grout material is placed vertically from the grout port at the lower end of the grout sleeve so that it may appear that the upper end of the sleeve is not filled, or the bottom leakage leads to the upper end of the cavity defect. The artificial defects should also be set at the upper end of the grout sleeve. Figure 1 is the schematic diagram of the sleeve with different grouting compactness and with two kinds of defects. Table 1 contains the main technical indicators of the grout sleeve. 

Therefore, six grouting compactness specimens with two grouting defects were prepared in this paper with 12 working conditions and two specimens for each condition for a total of 24 specimens. The reinforcement is connected to both ends of the sleeve before grouting and anchored through the reinforcement plug to avoid slurry leakage and eccentricity. The compactness of the specimen is controlled by filling the corresponding volume of grout material and the actual grouting compactness *F* is calculated using Formula (1).

(1) $$F=\frac{V_{1}}{V_{2}}\times 100\%$$

where *V*_1_ is the volume of actual grout and *V*_2_ is the inner volume of unfilled sleeve, which is measured by the equivalent method after connecting steel bars at both ends.

### 2.2. Test Rigs and Methods

The signal-receiving equipment in this experiment is the third generation fully digital system Sensor Highway III of Physical Acoustics Corporation (PAC). The acoustic emission sensor selects PK15I with a resonant frequency of 150 kHz to pick up the test signal. The broadband of the sensor is in the frequency range of 80–200 kHz. The acoustic emission detection parameters are shown in Table 2.

**Figure 2:**
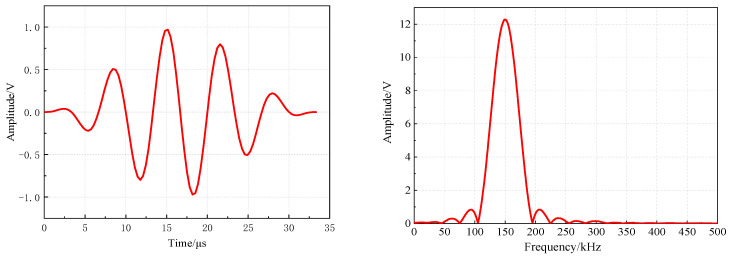
Time-domain waveform and spectrum of the excitation signal.

The signal generator is an AE-MAIN arbitrary waveform generator system. The sinusoidal signal modulated by Hanning window can make the energy concentrated in the center frequency attachment, suppress the dispersion and increase the propagation distance. Therefore, the excitation signal adopts a five-cycle sinusoidal signal with a central frequency of 150 kHz and an amplitude of 1 V. The excitation signal emission sensor is R3α. The expression of the excitation signal is (2). Figure 2 is the time domain waveform and spectrum of the excitation signal.

(2) $$F\left(t\right)=\{\begin{array}{cc} Asin\left(2\pi f\right)\left[1-\cos\left(\frac{2\pi f}{N}t\right)\right] & 0\leq t\leq \frac{N}{f}\\ 0 & t>\frac{N}{f} \end{array},$$

where *A* is the excitation signal amplitude, *A* = 1 V; *f* is the excitation signal center frequency, *f* = 150 kHz; and *N* is the excitation function period, *N* = 5. 

**Figure 3:**
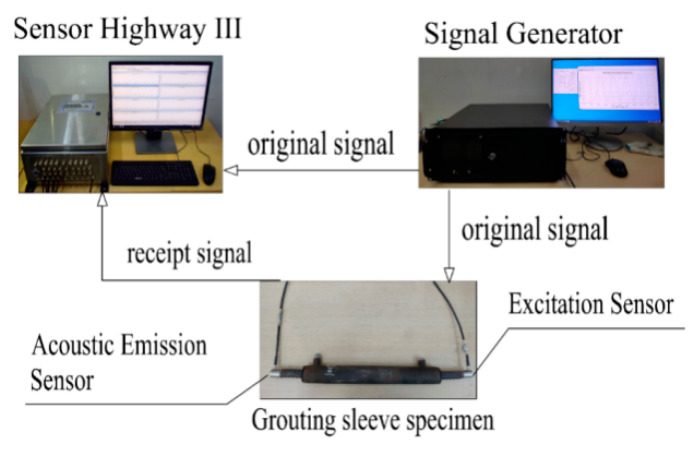
Test device.

In the experiment, the five-period sinusoidal pulse wave is emitted at one end of the specimen and then the acoustic emission sensor is used to receive the signal at the other end of the specimen. The arrangement of the two sensors is shown in Figure 3. The vacuum grease is used to couple the sensor and the specimen to improve the energy conversion efficiency of the sensor and reduce the signal loss and distortion. Each specimen was tested 5 times with an interval of 5 s.

## 3. Basic Analysis Methods

One of the most essential goals of acoustic emission technology is to detect the damage through the analysis of the acoustic emission source. Waveform analysis is the most accurate analysis method of acoustic emission signal in theory. Wavelet analysis has the function of noise separation and weak signal extraction, and its time-frequency analysis ability has great advantages in dealing with non-stationary random signals. The existing research [33,34] shows that the wavelet packet energy spectrum will produce significant changes due to the emergence of grout defects, and the grout defect evaluation index of guided wave energy attenuation is established. Therefore, the algorithm has potential in detecting sleeve grout defects. 

### 3.1. Wavelet Packet Analysis

Wavelet packet analysis [35] is a better signal time-frequency decomposition method. It can decompose the low frequency and high frequency parts of the signal at the same time. It belongs to the band-pass filter. According to the characteristics of the decomposed signal, the corresponding frequency band can be adaptively selected to match the signal spectrum and the time-frequency resolution is finer. 

**Figure 4:**
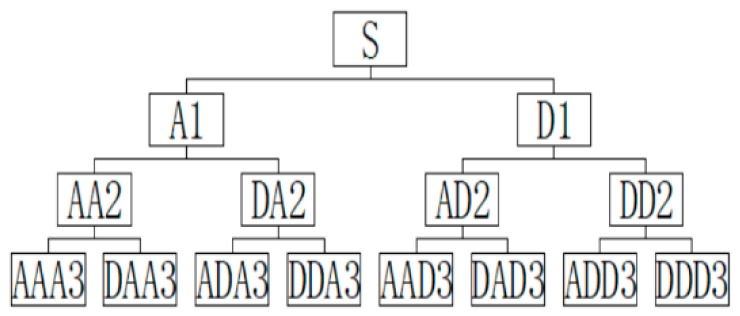
Three-level wavelet packet decomposition node diagram.

Each time the original signal is decomposed by wavelet packet, two sub-bands of low frequency and high frequency are obtained. Taking the three-layer wavelet packet decomposition as an example, the sub-signals of 2^3^, namely, eight different frequency bands, can be obtained in the third layer, as shown in Figure 4.

Different from the standard Fourier transform, wavelet packet analysis has a variety of wavelet functions that can be selected. The wavelet basis function with good temporal compactness, smoothness and symmetry should be selected in acoustic emission signal processing. The Symlets wavelet function is an approximately symmetric wavelet function proposed by Ingrid Daubechies, which has better symmetry, i.e., it can reduce the phase distortion when analyzing and reconstructing the signal to some extent. Symlets function is usually denoted as symN (N = 2, 3,..., 8). Since the symN wavelet function has a strong localization capability in the time and frequency domains in the processing of digital signals [36,37], the sym8 wavelet function was chosen for the processing of acoustic emission signals in this experiment.

In multi-scale decomposition of acoustic emission signals, the decomposition level should be determined according to the principle that the high frequency components after decomposition can reflect the original characteristics of the signal in detail. If the decomposition level is too low, the frequency range of a frequency band will be too large, the frequency characteristics of the signal will not be clearly distinguished due to the frequency overlap and the information of each frequency component of the signal cannot be displayed. When the decomposition level is too large, the frequency components of the signal have been decomposed, the larger scale is meaningless and the calculation amount will increase exponentially. Generally speaking, the decomposition scale is related to the main frequency band and the sampling frequency of the collected signal. The calculation formula is [38]:

(3) $$j\leq log_{2}\frac{f_{s}}{100}$$

The sampling frequency set in this paper is *fs* = 1 MHz. Through Equation (3) calculation, the decomposition scale at *j* = 3 can reflect the main characteristics of the signal. Therefore, the subsequent wavelet packet decomposition uses the sym8 wavelet function to decompose the acoustic emission signal into three layers. 

After three-layer wavelet packet decomposition, the bandwidth of each frequency band is 62.5 kHz. It should be noted that the sequence of nodes and frequency bands of the signal after wavelet packet decomposition is not consistent because the wavelet packet decomposition is a downward sampling process, and the frequency spectrum sequence of all components after high-frequency filtering and downward sampling is reversed once, resulting in the spectrum being arranged in the order of the Gray code. In order to make the high-resolution image, the nodes were grouped according to the frequency from low to high. The node order and range of the reconstructed signal at the last layer are shown in Table 3.

### 3.2. Evaluation Index of Grout Saturation

The process of constructing grouting compactness index by wavelet packet decomposition of acoustic emission signal energy ratio of each frequency band is as follows:

(1) After the acoustic emission signal *S* is decomposed by N-layer wavelet packet, the sub-signals in 2*^N^* frequency bands will be obtained at the N-layer, forming the signal set $\left\{X_{1},X_{2},X_{3},\ldots ,X_{2^{N}}\right\}$, in which one sub-signal is:
  (4) $$X_{j}=\left[x_{j,1},x_{j,2},x_{j,3},\ldots ,x_{j,m}\right]$$
  In the formula: *m*—sampling length; *j*—frequency band serial number, $j=1,2,3,\ldots ,2^{N}$.
(2) Assuming that the energy of one of the sub-signals after decomposition of the response signal under *i* condition is
  (5) $$E_{i,j}=X_{j}{}^{2}=x_{j,1}^{2}+x_{j,2}^{2}+x_{j,3}^{2}+\ldots +x_{j,m}^{2}\;$$
  where *i*—condition code.
  Then the wavelet packet energy of each frequency band formed by N-layer wavelet packet decomposition of response signal *S* under *i* condition can be expressed as
  (6) $$E_{i}=\left[E_{i,1},E_{i,2},E_{i,3},\ldots ,E_{i,2^{N}}\right]$$
(3) Let *EP_i_* be the sum of wavelet packet energies of each frequency band obtained by decomposition of response signals
  (7) $$EP_{i}=E_{i,1}+E_{i,2}+E_{i,3}+\ldots +E_{i,2^{N}}\;$$
  Then, the proportion of wavelet packet energy in each frequency band is
  (8) $$ER_{i}=\left[e_{i,1},e_{i,2},e_{i,3},\ldots ,e_{i,2^{N}}\right]$$
  where $e_{i,1}=\frac{E_{i,1}}{EP_{i}}\;,e_{i,2}=\frac{E_{i,2}}{EP_{i}},e_{i,3}=\frac{E_{i,3}}{EP_{i}},\ldots ,e_{i,n}=\frac{E_{i,2^{N}}}{EP_{i}}$
(4) Assuming that the response signal decomposition wavelet packet energy ratio of 100%, sleeve grouting compactness is
  (9) $$ER_{h}=\left[e_{h,1},e_{h,2},e_{h,3},\ldots ,e_{h,2^{N}}\right]$$
  where *h* means healthy.

The wavelet packet energy ratio of sleeve response signal with grouting compactness less than 100% is

(10) $$ER_{i}=\left[e_{i,1},e_{i,2},e_{i,3},\ldots ,e_{i,2^{N}}\right]$$

The compactness index (*FI*) based on wavelet packet energy ratio can be defined as:

(11) $$FI=\sqrt{\sum_{u=1}^{n}{\left(\frac{e_{i,u}-e_{h,u}}{e_{h,u}}\right)}^{2}}$$

The grouting compactness index (*FI*) screened the frequency band obtained by wavelet packet decomposition in the calculation and selected the frequency band with signal energy concentration in order to eliminate clutter interference and highlight the difference between the grouting compactness response signal and the grout defect response signal. According to the filling degree index, the filling degree of sleeve is judged.

## 4. Test Results and Analysis

### 4.1. Characteristic Parameter Analysis of the Acoustic Emission Signal

In order to analyze the propagation characteristics of acoustic emission signals in different grouting plumpness sleeves, the amplitude, energy and count of acoustic emission signals are selected to analyze the grouting compactness of sleeves. The test results are shown in Table 4.

#### 4.1.1. Acoustic Emission Signal Waveform Analysis

**Figure 5:**
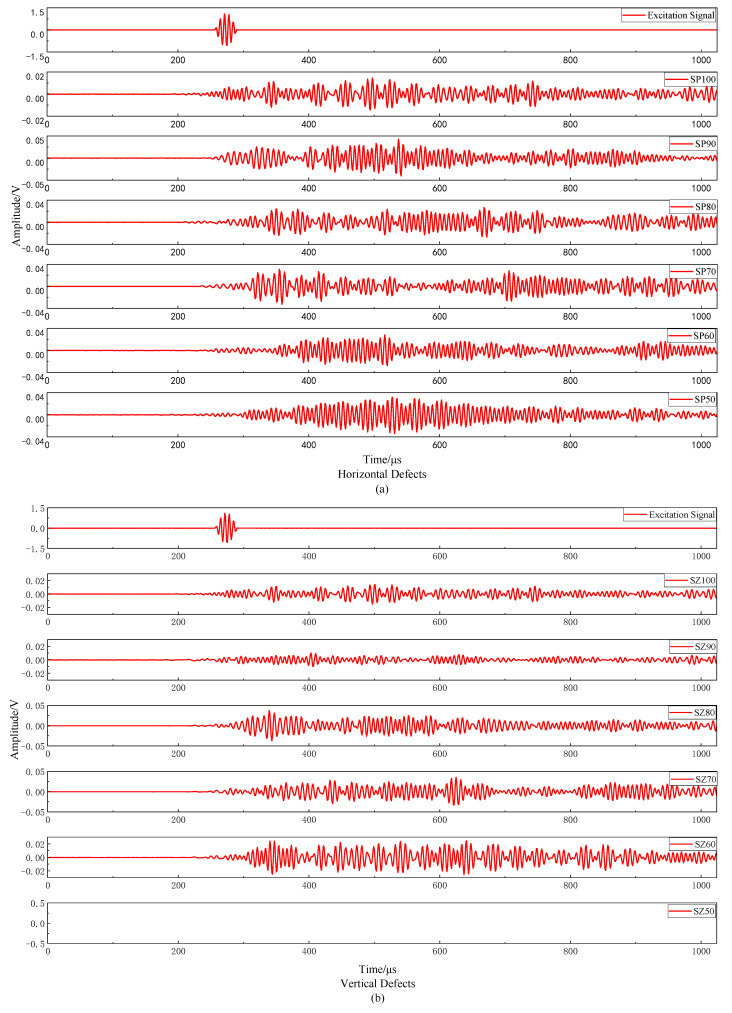
Receiving waveform of each group of specimens.

Figure 5 shows the waveform of the response signal of each grout sleeve specimen. The specimen with 50% saturation of the vertical defect cannot effectively connect the steel bars on both sides because of too little grout, which interferes with the acoustic emission instrument receiving the response signal on the other side of the steel bar. Therefore, the specimen of SZ50 needs to be removed in the subsequent analysis. This also shows that there is a situation in which sensors are arranged on both sides of the reinforcement because the grout amount is too small and the elastic wave cannot be transmitted from the excitation end to the receiving end, resulting in the acoustic emission instrument not being able to receive the response signal. After eliminating instrument failure and operation error, the response signal cannot be received, which indicates that the sleeve must have grout defects that seriously affect the connection performance of steel bars. 

The excitation sensor converts the excitation signal from the electrical signal to the displacement and acts on the specimen in the form of elastic wave. When the elastic wave propagates on the pure steel section, the kinetic energy of the elastic wave changes into internal energy due to the internal friction of the particles in the medium, which causes energy attenuation, that is, scattering attenuation. In the sleeve section, in addition to scattering attenuation, there is also energy loss caused by the diffusion of the elastic wave from the steel bar to the grout material, that is, diffusion attenuation. Therefore, it can be found that the attenuation of the response signal is very large and the law between the overall waveform and the saturation is not obvious. It is difficult to judge the saturation change by the change of the overall waveform.

#### 4.1.2. Amplitude Analysis of Acoustic Emission Signal

**Figure 6:**
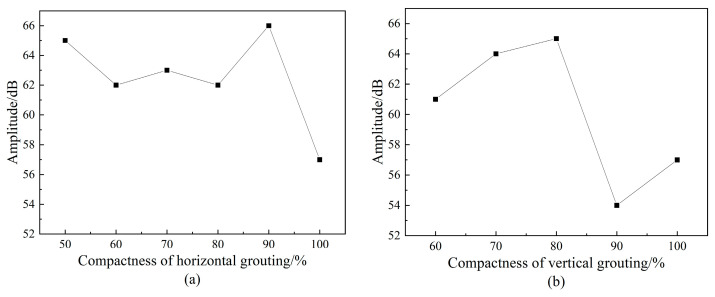
Amplitude of acoustic emission signals with different grout saturation.

Amplitude refers to the maximum peak voltage of an acoustic emission signal waveform, which is commonly used to characterize the type, strength and attenuation of an acoustic emission source and is usually in dB. Figure 6 is the comparison chart of the acoustic emission signal amplitude with different saturation under two kinds of defects. It can be found that the acoustic emission signal amplitude does not reflect the influence of saturation change on it. The main reason is that the acoustic emission amplitude is the maximum amplitude value of the waveform, and the maximum waveform amplitude is not completely through the whole specimen; it is likely to be the reflection wave of the defect or other clutter superposition. A study [39] found that the void degree of the sleeve can be distinguished by the first wave amplitude of the stress wave. However, it can be seen on Figure 5 that the waveform of the acoustic emission signal has the attenuation and superposition of the wave packet, resulting in a great difference from the waveform of the excitation signal, and it is difficult to distinguish the first wave from the waveform with distortion. Therefore, it is also difficult to evaluate the grouting compactness of the sleeve by the amplitude parameters of the acoustic emission signal.

#### 4.1.3. Acoustic Emission Signal Energy Analysis

**Figure 7:**
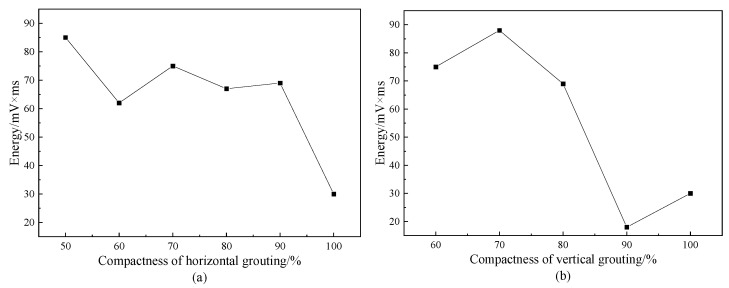
Acoustic emission signal energy with different grout saturation.

On Figure 7, we can see that the variation curve of acoustic emission energy parameters does not show regularity and cannot reflect the grouting compactness in the sleeve. Acoustic emission energy can reflect the relative energy or intensity of acoustic emission events, which is often used as the evaluation standard for acoustic emission tests of materials or structures. Numerically, it refers to the area under the detection envelope of the acoustic emission signal, which is expressed as the integral of voltage to time. Therefore, it can be seen that the acoustic emission energy and the acoustic emission amplitude have a certain connection within a certain time length of the collected waveform. Therefore, the curves of energy and amplitude are relatively similar and the identification of different saturation levels also has the same problem.

#### 4.1.4. Analysis of the Count of the Acoustic Emission Signal

**Figure 8:**
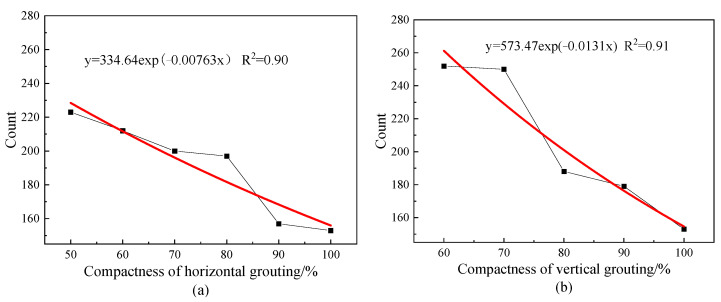
The counts of acoustic emission signals with different grout saturation.

The number of oscillations that the signal passes through the set threshold is the count, which is suitable for both burst and continuous signals, so it is widely used to evaluate acoustic emission activity. It can be found on Figure 8 that the count will follow the grout saturation and will show a downward trend with the increase of the saturation, indicating that the grouting compactness will affect the activity of acoustic emission. Therefore, we use the exponential function to fit it and R^2^ is 0.9, indicating that the fitting degree is good. From the fitting function, it can be found that the decline rate of the vertical defect is slightly larger than that of the horizontal defect, indicating that the count is greatly affected by the vertical defect. Based on the obtained law of test samples, the count parameters can reflect the change of grouting compactness to a certain extent.

From the above analysis, it can be seen that the change of most acoustic emission characteristic parameters is not obvious enough to distinguish the change of grout saturation. Further analysis, conversion and verification are needed to obtain more reasonable parameters to identify the change of grout saturation.

### 4.2. Acoustic Emission Signal Wavelet Analysis

#### 4.2.1. Wavelet Packet Energy Proportion Analysis

**Figure 9:**
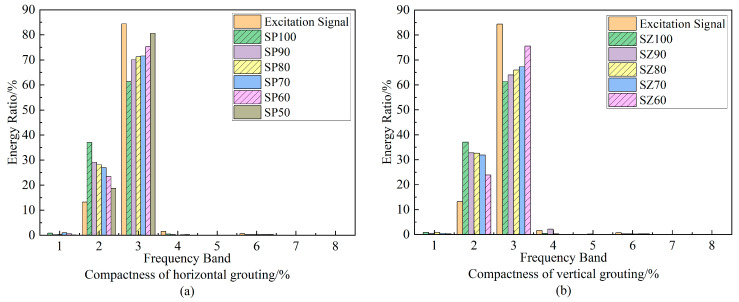
Relationship between wavelet packet energy proportion and grout saturation.

The sub-band energy ratio after wavelet packet decomposition can enlarge the damage effect and effectively distinguish structural damage. Therefore, we used the sym8 wavelet function to decompose the signal received by the acoustic emission instrument into three layers of wavelet packet, and the energy of each frequency band component with different grouting compactness is accounted for as shown in Figure 9. Figure 9 shows that the excitation signal has the largest energy proportion in the third frequency band of approximately 125–187.5 kHz, which conforms to the characteristics of the center frequency 150 kHz five-wave signal. It shows that it is reasonable and effective to select the sym8 wavelet basis function to decompose the original signal into three layers of wavelet packet.

The response signals received by the acoustic emission instrument are mainly concentrated in the second and third node bands, i.e., approximately 62.5–187.5 kHz. The sum of the energy ratios of these two bands is more than 90%, which is far more than the sum of the energy ratios of other bands. Moreover, frequency band 2 and frequency band 3 are sensitive to the change of filling degree, which can intuitively show the change law of filling degree and wavelet packet energy ratio of the detection signal.

With the increase of grout saturation, whether with horizontal defects or vertical defects, there will be a decrease in the energy proportion of frequency band 3 and an increase in the energy proportion of frequency band 2. This shows that the composition of approximately 125–187.5 kHz in the elastic wave is more prone to dispersion in the sleeve with high grout saturation, resulting in large loss. The appearance of defects in the sleeve makes the elastic wave reduce the loss and does not differ from the distribution of grout materials. 

By comparing the energy proportion changes of band 2 and band 3 of horizontal defects and vertical defects, it can be found that with the decrease of saturation, the change of energy proportion under vertical defects is more obvious than that of horizontal defects, indicating that the approximately 125–187.5 kHz part of the elastic wave is sensitive to the change of vertical grout defects.

#### 4.2.2. Analysis of Grouting Compactness Index

**Figure 10:**
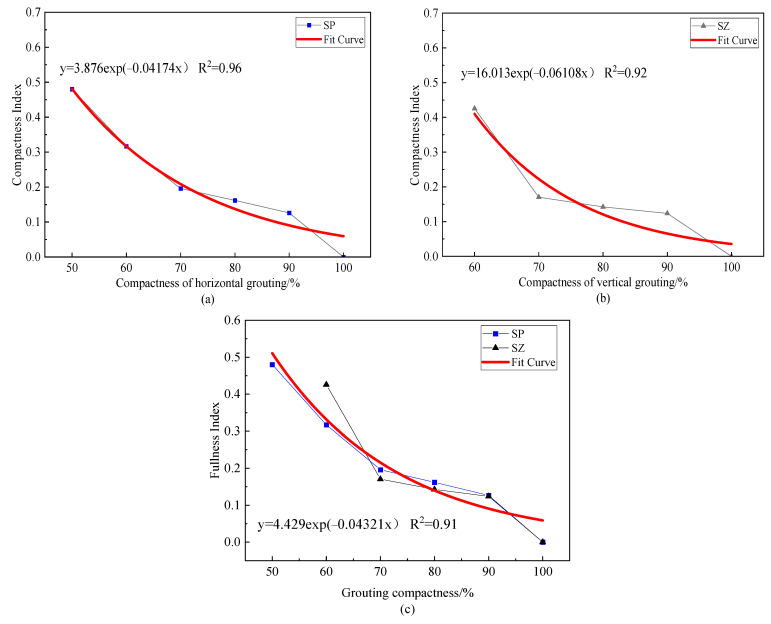
Index map of grout saturation.

The change of grouting compactness will affect the energy attenuation in the process of elastic wave propagation, resulting in the change of the energy proportion of each frequency band after wavelet packet decomposition. In order to compare the energy change and frequency shift of each frequency band of the received signal under different grout saturation, this paper constructs the saturation index according to the difference of the energy proportion of each frequency band after wavelet packet decomposition of the response signal of the grout full specimen and the grout incomplete specimen to quantitatively describe the grout defect degree of the grout sleeve. Using Formula (11), the relationship between the saturation index and the saturation of each group of specimens was calculated and the results are shown in Figure 10.

From Figure 10, it can be concluded that the compactness index decreases significantly with the increase of the filling degree and the decreasing trend is accelerated. By fitting the exponential function, two models describing the relationship between filling degree and compactness index are obtained: when the horizontal distribution of grout material is y = 3.876exp (−0.0417x) and when the vertical distribution of grout material is y = 16.013exp (−0.06108x), the fitting curve can be seen in the red curve in Figure 10a,b. R^2^ are 0.96 and 0.92, respectively, indicating that the compactness index is in line with the exponential decay trend with the increase of the filling degree. This is because when there are grout defects inside the grout sleeve, the contact area between the steel bar and the grout material becomes less during the propagation of the elastic wave inside the steel bar, so the energy leaked to the grout material and the outer wall of the sleeve is reduced and the redistribution of the frequency band energy is affected. The concrete manifestation is that the difference between the signal of the full grout specimen and the signal of the defect specimen increases and the saturation index value increases.

The observation shows that the two models are similar, that is, the attenuation rate of the filling degree index of the vertical defect is not much different from that of the horizontal defect, so we expect that the change of filling degree index FI has little relationship with the distribution of grout material. Therefore, we combined and fitted the FI values under the two distribution forms by exponential function and obtained the relationship model between FI and grouting compactness as follows: y = 4.429exp (−0.04321x). The fitting curve is shown as the red curve in Figure 10c and its R^2^ = 0.91, indicating that this model can accurately reflect the change law of FI with grout saturation. Therefore, we think that the saturation index is almost unaffected by the distribution form of the grout material.

In practical engineering applications, several groups of standard grout sleeve models with different filling degrees can be prepared in advance in the laboratory according to the requirements of field construction technology and materials, in which 100% grout sleeve must be included and the grout operation must be performed according to the specification requirements. Then, the model was tested according to the proposed method and the proportion of wavelet packet energy and the fullness index were calculated to draw the relationship curve between FI and grout saturation. At the same time, the unknown sleeve grouting compactness at the construction site is tested and then the FI of the specimens to be tested is calculated according to the wavelet packet energy ratio obtained from the prefabricated 100% grouting compactness model. Finally, the saturation of the unknown specimen can be calculated by substituting the FI of the test specimen into the relationship curve between compactness index and grouting compactness drawn by the prefabricated standard grout sleeve model.

## 5. Conclusions

In this paper, the signal generator is used as the excitation source and the acoustic emission instrument is used to detect the two common grout sleeves with different filling degrees. In addition to the comparison of acoustic emission signal characteristic parameters, the wavelet packet decomposition is used to analyze the acoustic emission waveform, and the filling degree evaluation index based on wavelet packet energy ratio is proposed, which verifies the effectiveness of the proposed method. The main conclusions are as follows:

1. When the sensors are arranged on both sides of the steel bar, the response signal cannot be received under the premise of ensuring that the instrument has no fault and the operation is correct, which indicates that the sleeve must have grout defects that seriously affect the connection performance of the steel bar.
2. The amplitude and energy of AE signal characteristic parameters are susceptible to the interference of steel bars and grout materials due to the excitation signal, and their consistency with the change of grouting compactness is not obvious. The count can reflect the difference between different grouting compactness to a certain extent. With the increase of saturation, the count shows an exponential downward trend and the downward rate is affected by the shape of the grout material. The downward rate of vertical defects is slightly larger than that of horizontal defects.
3. Whether the shape of the grout material is horizontal or vertical, the increase of grouting compactness will lead to a decrease of energy proportion in the range of approximately 125–187.5 kHz and an increase of energy proportion in the range of approximately 62.5–125 kHz, indicating that the increase of saturation will affect the transformation of high-frequency components in the signal to low-frequency components, but the vertical defect will accelerate the transformation of high-frequency components.
4. Based on the energy proportion of wavelet packet decomposition, the evaluation index FI of grouting compactness is proposed to quantitatively evaluate the size of saturation. The results show that the FI value of the saturation index decreases significantly with the increase of saturation, and the decreasing trend is an exponential function, indicating that the specimen with defects can greatly reduce the attenuation of energy in the signal.
5. The change in the FI value has a high consistency with the defect size and has little relationship with the distribution form of grout material in the sleeve. Therefore, the filling saturation index FI under the two distribution forms of grout material can be combined and fitted, and the general law of FI and grouting compactness can be further obtained.

By calculating the grouting compactness evaluation index, the proposed detection method based on acoustic emission technology can basically realize the recognition of grout saturation, which has potential in the grouting compactness detection of reinforced sleeve grout connection components. In addition, for the identification of grouting compactness under different conditions, such as different diameter reinforcement, different materials sleeve, different forms of sleeve, etc., the relationship between FI and grouting compactness needs further research and verification.

## Figures and Tables

**Table 1:** Main technical indexes of specimens.

Specimen Number	Defect Type	Saturation of Grout (%)	Sleeve Type	Steel Bar Diameter (mm)	Anchorage Length (mm)
SP100	Horizontal defects	100%	GTZQ4-20A	20	160
SP90		90%			
SP80		80%			
SP70		70%			
SP60		60%			
SP50		50%			
SZ100	Vertical defects	100%	GTZQ4-20A	20	160
SZ90		90%			
SZ80		80%			
SZ70		70%			
SZ60		60%			
SZ50		50%			

**Table 2:** Parameters setting of acoustic emission acquisition.

Pre-Gain (dB)	Threshold (dB)	Sampling Rate (MSPS)	Sampling Length	Sampling Time (ms)	PDT (μs)	HDT (μs)	HLT (μs)
26	35	1	1k	1.024	100	200	300

Where PDT is peak definition time, HDT is hit definition time, and HLT is hit locking time.

**Table 3:** Frequency band range of layer 3 nodes after wavelet packet decomposition.

Frequency Band Number	Node Number	Frequency Range (kHz)
1	7	0~62.5
2	8	62.5~125
3	10	125~187.5
4	9	187.5~250
5	13	250~312.5
6	14	312.5~375
7	12	375~437.5
8	11	437.5~500

**Table 4:** Summary of acoustic emission signal characteristic parameter test results.

Saturation of Grout	Amplitude (dB)		Energy (mV × ms)		Count	
	Horizontal	Vertical	Horizontal	Vertical	Horizontal	Vertical
100%	57	57	30	30	153	153
90%	66	54	69	18	157	179
80%	62	65	67	69	197	188
70%	63	64	75	88	200	250
60%	62	61	62	75	212	252
50%	65	-	85	-	223	-

## Data Availability

The data used to support the findings of this study are included within the article.
